# Fabrication of High-Aspect-Ratio 3D Hydrogel Microstructures Using Optically Induced Electrokinetics

**DOI:** 10.3390/mi7040065

**Published:** 2016-04-12

**Authors:** Yi Li, Sam H. S. Lai, Na Liu, Guanglie Zhang, Lianqing Liu, Gwo-Bin Lee, Wen Jung Li

**Affiliations:** 1 Department of Mechanical and Biomedical Engineering, City University of Hong Kong, Kowloon, Hong Kong, China; yili58-c@my.cityu.edu.hk (Y.L.); samlai5-c@my.cityu.edu.hk (S.H.S.L.); 2 State Key Laboratory of Robotics, Shenyang Institute of Automation, Chinese Academy of Sciences (CAS), Shenyang 110016, China; liuna@sia.cn (N.L.); lqliu@sia.cn (L.L.); 3 Shenzhen Academy of Robotics, Shenzhen 518000, China; glzhang@szarobots.com; 4 Department of Power Mechanical Engineering, National Tsinghua University, Hsinchu 300, Taiwan; gwobin@pme.nthu.edu.tw

**Keywords:** optically induced electrokinetics, polymer microfabrication, hydrogel microstructures, 3D polymer structures

## Abstract

We present a rapid hydrogel polymerization and prototyping microfabrication technique using an optically induced electrokinetics (OEK) chip, which is based on a non-UV hydrogel curing principle. Using this technique, micro-scale high-aspect-ratio three-dimensional polymer features with different geometric sizes can be fabricated within 1–10 min by projecting pre-defined visible light image patterns onto the OEK chip. This method eliminates the need for traditional photolithography masks used for patterning and fabricating polymer microstructures and simplifies the fabrication processes. This technique uses cross-link hydrogels, such as *poly(ethylene glycol) (PEG)-diacrylate* (PEGDA), as fabrication materials. We demonstrated that hydrogel micropillar arrays rapidly fabricated using this technique can be used as molds to create micron-scale cavities in PDMS (*polydimethylsiloxane*) substrates. Furthermore, hollow, circular tubes with controllable wall thicknesses and high-aspect ratios can also be fabricated. These results show the potential of this technique to become a rapid prototyping technology for producing microfluidic devices. In addition, we show that rapid prototyping of three-dimensional suspended polymer structures is possible without any sacrificial etching process.

## 1. Introduction

The demand for micro-/nano-scale MEMS components and devices has been rapidly increasing in electronics, optics, medicine, bioengineering, automotive and optical communication-related research and products [1,2]. Some specific applications include organ implants, rapid diagnostic devices, wearable health-monitoring devices, micro-scale gyroscopes and micro-lenses. Moreover, microfluidic chip components such as micro heaters, micro-scale pumps, valves and mixing devices also require MEMS fabrication technology. Recently, there has been increasing research interest in developing micro-/nano-scale 3D (three-dimensional) fabrication technologies such as photolithography, colloidal epitaxy with masking, and direct writing [3]. The micro-/nano-scale lithography technologies include X-ray lithography, extreme ultraviolet lithography, nanoimprint lithography, scanning probe lithography, and thermochemical nanolithography [4,5]. Using these technologies, 3D micro-/nano-scale MEMS structures have been fabricated, including 3D polymer scaffolds and complex 3D polymer vascular networks [6]. Among these technologies mentioned above, direct writing offers flexibility in materials selection, capability for rapid prototyping, and micro-/nano-scale precision with low operation and maintenance costs; hence, it is becoming one of the most promising approaches for 3D micro-/nano-scale fabrication. Even though this technique shows promise in rapid prototyping fabrication applications, the path scanning process inherent in this technique still imposes a x-y plane fabrication speed limitation of ~5 mm/s with a limited lateral resolution of 150 µm for a nozzle-based scanning method [7]. We should note that the OEK-based technique reported in this paper has already demonstrated patterns with lateral resolution of ~5 µm. Moreover, the opto-electrokinetics-based micromanipulation techniques have been reported since 2000. For example, researchers have shown that the particles form colloidal crystals could be assembled using optically tunable patterns to control electrokinetics forces [8,9]. However, the manipulation force was so weak that the whole experiment process took ~2 h [8]. This technique was improved by Chiou *et al.*, who realized massively manipulated single cells and micro-particles in OEK chips [10]. We should note here that our system is similar to the system originally reported by Chiou *et al.* in [10] Other researchers also have demonstrated using opto-electrokinetics techniques that introduce changes in the organic thin film local area surface hydrophilicity, and hence enabled the manipulation of small particles [11].

In this paper, we present our recent work in using a light pattern induced electrokinetics field to fabricate 3D polymer microstructures in a microfluidic environment. In our previous research [12], basic micron-scale *poly(ethylene glycol) (PEG)-diacrylate* (PEGDA) hydrogel features approximately 3 µm in height were fabricated using an optically-induced electrokinetics (OEK)-based platform. The principle of the OEK can be described as follows. When an AC bias voltage is applied across the top ITO glass and the bottom OEK chip, which is made from an ITO glass coated with an a-Si:H layer, a small electric potential drops across the liquid layer and an uniform electric field is produced in the liquid layer. When a projected light pattern is illuminated onto the photosensitive a-Si:H thin film, the conductivity of the a-Si:H thin film will increase from 10^−11^ to 10^−5^ S/m. This local conductivity change on the a-Si:H thin film will cause a localized non-uniform distribution of the electric field, and hence the projected light pattern acts as a virtual localized electrode. The particles in between the two glasses will experience OEK force and move towards or away from the virtual localized electrode [12]. We have further investigated the OEK-based polymerization technology and show in this paper that a single-step fabrication process can be used to rapidly prototype polymer microfluidic components and create 3D suspended polymer microstructures. We should note here that PEGDA is a commonly used biomaterial and could be polymerized with free radicals to form long-chain molecular networks with adjustable mechanical properties when exposed to an ultraviolet light source as shown by others in the past [13]. In this paper, we demonstrate the PEGDA polymerization process when it is exposed to a non-uniform AC electrical field and a non-ultraviolet light source in an OEK chip. This technique can be used to rapidly fabricate micron-scale PEGDA structures within a typical 60–120 s. We also demonstrate the fabrication process of microtubes and 3D microstructures with undercut features using the aforementioned technique. Different from the existing photolithography technique, this method uses hydrogel materials like PEGDA as pre-polymer. DI water was used as the developing solution during the hydrogel mold fabrication process. Thus, this fabrication process is an inexpensive, simple, fast and environmentally friendly micro-/nano-scale features patterning technique. In addition to the micro-/nano-scale rapid prototyping capability, this technology could potentially be used for micro-particle trapping and manipulation, which is also reported in this paper.

## 2. Materials and Methods

### 2.1. OEK System

**Figure 1:**
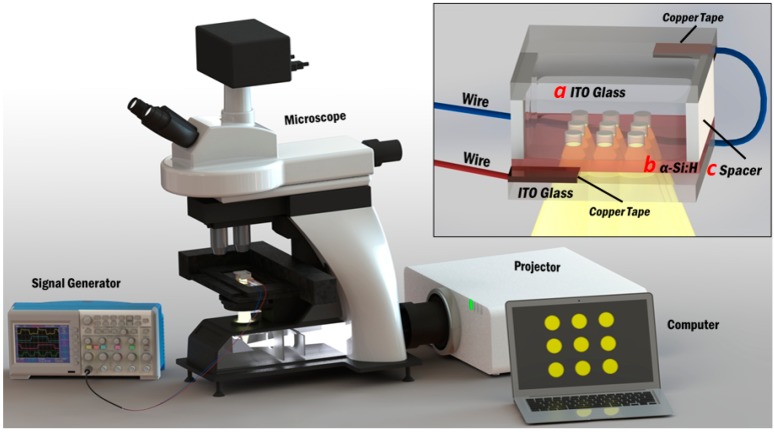
Illustration of the experimental system and the OEK chip (shown in inset). The experimental system consists of a computer, a signal generator, a display projector, and a microscope; the inset figure shows structural details of the different OEK chip layers. (a) ITO glass thickness is 2 mm; (b) a-Si:H layer thickness is 200 nm; (c) spacer height is 50 μm.

The OEK integrated research platform used for the experiments is shown in Figure 1. The system includes two main modules: the OEK chip module and the peripheral supporting module. The OEK chip module includes three parts, as shown in the inset of Figure 1: (1) a glass slide (3 cm × 3 cm) cover coated with an indium tin oxide (ITO) thin film, which serves as the top electrode; (2) a microfluidic chamber made with PDMS (*polydimethylsiloxane*) thin film in which the PEGDA solution is contained; (3) an OEK glass substrate (3 cm × 3 cm), which has a thin layer of a-Si:H film on top of glass coated with ITO. The gap between ITO glass and OEK chip is 50 μm; ITO and OEK chip thickness are 2 mm respectively. The peripheral supporting module includes a microscope, a computer-controlled projector, and a signal generator.

The basic process of assembling the three components of the OEK chip module is described below. The first step is to clean the top ITO glass and bottom OEK glass substrates by immersing them in 95% *v*/*v* ethanol (Sigma-Aldrich Co. LLC., St. Louis, MO, USA) and sonicating them for 30 min at a frequency of 59 kHz (KS-8893, Ningbo Haishu Kesheng Ultrasonic Equipment Co., Ltd., Ningbo, China). Then, they are blown dry with nitrogen gas. Subsequently, the top ITO glass and OEK glass substrates are assembled with a spin-coated and patterned PDMS spacer layer (50 μm thick), placed between the two glass substrates to form a microfluidic “OEK chamber” into which the PEGDA pre-polymer solution can be injected. The OEK chip module is loaded on a two-dimensional high precision translation stage, as shown in Figure 1. An AC electrical signal generated by a signal generator (Agilent 33522A, Agilent Technologies, Inc., Santa Clara, CA, USA) with an amplifier (TG4001, TTI, Huntingdon, Cambridgeshire, UK) is applied between the top ITO glass and bottom OEK glass electrodes. Then, a series of programmable light patterns, generated by a commercially available computer software (Microsoft PowerPoint 2010, Microsoft Co., Redmond, WA, USA), are projected from an LCD projector (PT-EW630, Panasonic Co., Osaka, Japan) onto the OEK glass a-Si:H substrate through a custom-designed light condenser module (Eclipse Ti-E, Nikon Instruments Inc., Melville, NY, USA).

### 2.2. OEK-Induced Polymerization

Similar to the fabrication of photoresist-based structures, traditional PEGDA microstructure fabrication processes were based on photolithographic technique, meaning that an ultraviolet light source is essential to curing the solution of PEGDA mixed with photoinitiator. A lithographic mask is also necessary to define the predesigned microstructure patterns. By contrast, the photon-induced electrokinetics method presented in this paper could fabricate PEGDA microstructures without the use of ultraviolet light and masks. The mechanism of polymerizing the PEGDA hydrogel has been demonstrated in our previous work. That is, the electrons generated from the a-Si:H layer of the OEK chip by the photoelectric effect will combine with the hydrogen ions in a hydrogel solution and continue to reduce to hydrogen radicals. These hydrogen radicals will trigger the PEGDA polymerization chain reaction [14].

### 2.3. Micro-Scale Feature Characterization

**Figure 2:**
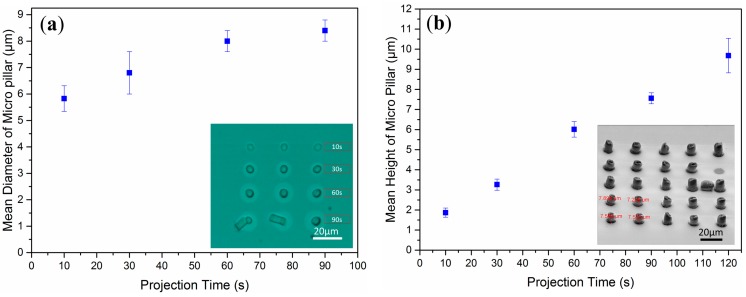
The relationship between physical size of hydrogel micro pillar structure and exposure time. (**a**) The dependence of hydrogel micropillar diameter on exposure time (3 pillars were measured for each data point). Inset: Optical image of a 4 × 3 pillar array showing that the micropillar aspect ratio increased as a function of exposure time. (**b**) Plot of the relationship between hydrogel micropillar height and light pattern exposure time. Each data point contains height measurements from 4 microstructures. Inset: eSEM image of a 5 × 5 micro pillar array. The light pattern projection time required to obtain the structures shown here was 90 s.

To determine the relationship between micro-feature geometry and exposure time experimentally, we fabricated micropillar arrays using the OEK-induced polymerization system. Figure 2a shows an optical image of a micropillar array and demonstrates the relationship between the diameters of patterned hydrogel pillars as a function of exposure time. We chose four different exposure times (10, 30, 60, 90 s) and measured the diameters of the PEGDA micropillars in the 4 × 3 array using an optical microscopy image, as shown in the inset of Figure 2a. From the optical images shown in the Figure 2a inset, the diameters of the PEGDA pillars grow as the exposure time increases. Initially, the hydrogel micropillar diameter was relatively small, less than the 10-μm diameter projected image spot size. As the exposure time increased, the diameter increased; however, the diameter size became stable after ~90 s.

Figure 2b shows the relationship between micropillar height and light pattern projection time. The hydrogel micropillar heights were measured using environmental scanning electron microscope (eSEM) images, as shown in the Figure 2b inset. Figure 2b shows a plot of the relationship between hydrogel micropillar height and light pattern projection time. The error bars in Figure 2a,b represent the standard deviation of the fabrication mean diameter and fabrication mean height of the micropillars, respectively. The measurement of the physical size of the micropillar is based on a commercial image processing software, so the error bars show the fabrication error for every sample group.

## 3. Results and Discussion

### 3.1. Effect of Solution Conductivity

**Figure 3:**
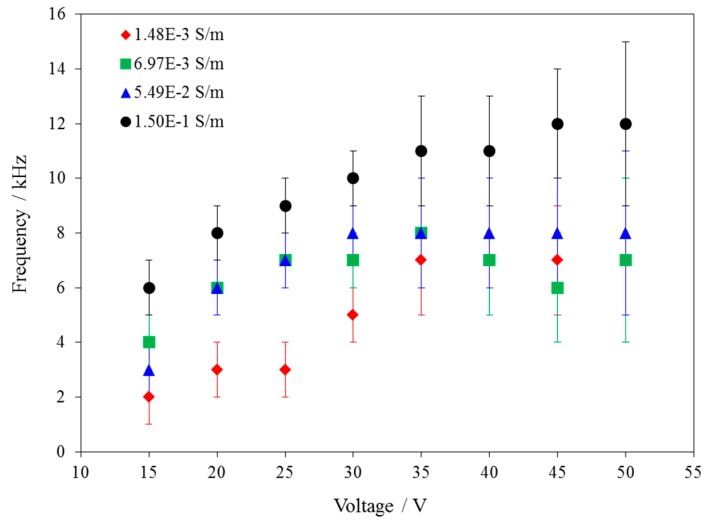
The required mean maximum frequency and voltage for hydrogel polymerization using PEGDA solutions with different polymer solution electrical conductivities. This plot indicates that as the electrical conductivity of the solution increases or the applied voltage increases, the frequency required for hydrogel polymerization also increases.

PEGDA polymerization on an OEK chip is controlled by the potential drop across the a-Si:H/hydrogel solution interface. There is a minimum potential, which can activate the polymerization process. Based on the equivalent circuit of the OEK chip [14], the interface potential will decrease with the increase of the AC frequency, but it will increase with the increase of AC voltage and solution conductivity. Therefore, at a given combination of conditions of AC voltage and solution conductivity, there is a maximum frequency below which all the AC electric field can activate the polymerization process. Figure 3 gives the mean experimental maximum frequency under different V_pp_ and solution conductivities. For example, for an applied voltage of 35 V_pp_ (the equivalent electric field value is 0.7 V_pp_/μm) and a solution conductivity of 1.48 × 10^−3^ S/m, all AC frequencies less than ~7 kHz are suitable to activate the PEGDA polymerization process. However, when the voltage decreases to 20 V_pp_ (the equivalent electric field value is 0.4 V_pp_/μm), the maximum frequency is ~3 kHz. The error bars in Figure 3 represent the standard deviation of the mean maximum frequency required for PEGDA polymerization. The PEGDA polymerization depends on the mean maximum frequency (the mean maximum frequency can be defined as the mean value of maximum frequency from the three experiments, which is shown as one data point in Figure 3), applied voltage, and conductivity of the PEGDA solution. For a single data curve, as the applied voltage is increased, the PEGDA polymerization mean maximum frequency also increased. If we fix the voltage, as the PEGDA solution conductivity increased, the PEGDA polymerization mean maximum frequency also increased. The curve clearly shows these relationships.

### 3.2. Mold Fabrication

**Figure 4:**
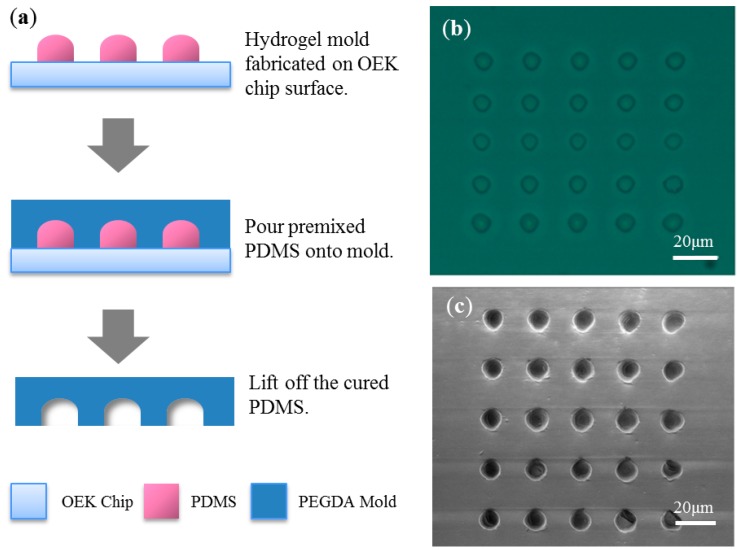
Illustration of the PDMS microcavity array fabrication process using a PEGDA mold fabricated by the OEK-based process. (**a**) Process flow schematic of the PDMS microcavity array fabrication. (**b**) Optical microscope image of a 5 × 5 PEGDA hydrogel micropillar array. Each micropillar was approximately 10 μm in diameter and approximately 5 μm in height. Optical pattern projection time is 60 s, apply voltage is AC 10 V_pp_, (the equivalent electric field value is 0.2 V_pp_/μm), frequency is 10 kHz. (**c**) eSEM image of the micron-scale cavities molded from the PEGDA hydrogel micropillar array. Each microcavity was approximately 10 μm in diameter and approximately 5 μm in depth.

These hydrogel micropillar arrays can also be used as molds for PDMS casting, with potential applications in microfluidics. This molding process is shown in Figure 4a. A PDMS (Sylgard 184, Dow Corning S.A., Seneffe, Belgium) elastomer solution was prepared by mixing prepolymer with a cross-linking agent at a weight ratio of 10:1. Air bubbles in the PDMS mixture were removed by placing the mixture under vacuum for 1 h. Then, the PDMS mixture was poured onto the fabricated hydrogel mold and cured in an oven at 65 °C for 2 h. After curing, the PDMS replica was peeled from the mold. Figure 4b shows an optical image of the micropillar array, and Figure 4c shows an eSEM image of the micro-cavity array corresponding to the micropillar mold.

The first advantage of the mold fabrication method depicted in Figure 4 is that the method is very simple compared with other existing molding processes. The photolithography-based mold creation process comprises substrate cleaning, spin coating, pre-baking, UV exposure, post-baking and resist developing. These steps usually require tens of minutes for completion, in addition to requiring the use of photolithographic masks. However, the hydrogel mold fabrication process described here can be completed in a few minutes and requires no lithographic masks. Moreover, this hydrogel mold fabrication process is an inexpensive method. In general, photolithography processes require UV exposure systems, spin-coating machines, and hot plates. By contrast, the hydrogel fabrication method described in this paper only requires a small visible light source. Photolithography processes are typically completed on silicon wafers using expensive photoresists, such as SU-8, KMPR, and AZ; each type of photoresist requires the use of a suitable developing solution. By contrast, the slide substrate and hydrogel used in the OEK-based method described here are inexpensive materials, and DI (deionized) water can be used as the “developing solution” during the hydrogel mold fabrication process, *i.e.* any unexposed hydrogel can be washed away by DI water. Thus, this fabrication process is an inexpensive, simple, fast and environmentally friendly patterning process.

However, one challenge of this novel process that needs to be overcome is the adhesion between the fabricated hydrogel structures and the substrate. To be used as a mold for subsequent casting procedures, firm adhesion of the hydrogel to the substrate is required. It was found that the water used to remove the unexposed hydrogel solution during the development step caused the polymerized hydrogel material to swell. However, the substrates, which were made of other materials such as glass, silicon and metal did not swell. These volume expansions mismatched in two different materials resulted in stress increased at the contact surface. Hence, the adhesion force between the hydrogel structures and the substrate was not sufficient to allow the hydrogel micropillars to serve as molds for creating micro-cavities in a PDMS substrate. We solved this problem by adding a thin hydrogel layer on top of the OEK chip surface after inducing the OEK-based polymerization process. A circle light pattern with a diameter bigger than diagonal length of micropillar array will project to the OEK chip. The exposure time is 1 s with same electrical parameters as micropillar fabrication. Then, a thin layer of polymerized PEGDA will attach itself to the micropillar array. This method will help to fix the microfeatures on top of a-Si:H.

### 3.3. Micro-Scale Tube Fabrication

**Figure 5:**
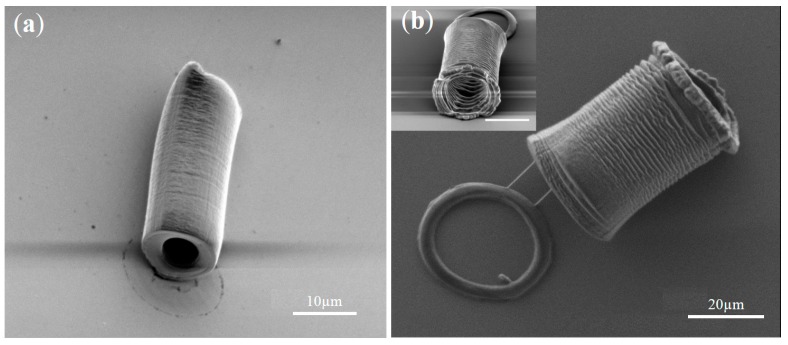
eSEM images of hydrogel microtubes. (**a**) A PEGDA hydrogel tube with a length of approximately 20 μm, an outer diameter of approximately 10 μm, and an inner diameter of approximately 5 μm. Optical pattern projection time is 200 s with applied voltage AC 10 V_pp_, the equivalent electric field value is 0.2 V_pp_/μm, and frequency is 10 kHz. (**b**) A PEGDA hydrogel tube with a length of approximately 35 μm, an outer diameter of approximately 25 μm, and an inner diameter of approximately 20 μm. Optical pattern projection time is 320 s, apply voltage is AC 10 V_pp_, the equivalent electric field value is 0.2 V_pp_/μm, and frequency is 10 kHz. The inset image is a tilted view of the PEGDA hydrogel tube, scale bar: 20 μm.

In addition to applying the OEK-induced hydrogel polymerization principle to fabricate hydrogel micropillars for molding microfluidic cavities, this method can also be applied to other 3D microstructures, as shown in Figure 5. We show that the OEK-induced hydrogel polymerization technique can be used to fabricate hydrogel tubes of different sizes. Such micro-tubes could be the basic building blocks to a complex vascular network. The generation of a complex 3D vascular network could then be used to engineer highly vascularized tissues and organs for regenerative medicine [15] and pharmacological screenings [16]. In general, the process described here could be used to fabricate functionalized microstructures with different sizes and aspect ratios on top of the same substrate.

### 3.4. Polystyrene Bead Trapping by a Hydrogel Tube

**Figure 6:**
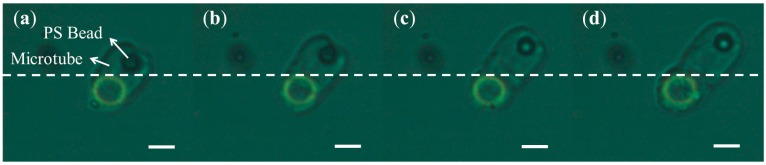
Optical images of a PS (*polystyrene*) bead trapped and manipulated by a hydrogel microtube structure. (**a**–**d**) Time-lapse images recorded the process of a PS bead been trapped by a hydrogel microtube (scale bar: 5 μm): (**a**–**c**) 5s interval; (**d**) after 180 s exposure time. Green ring is the projected light pattern. White dash line indicates the initial position of PS bead.

For applications in biomedicine, traditional cellular studies are conducted using large quantities of cells; consequently, the resulting measurements only indicate statistical cellular data. This approach may misinterpret the true physiological state of cells because it ignores the differences between individual cells. In the past few decades, single-cell analysis has become more important in biomedical research, and many cell entrapment or manipulation techniques have been explored. Existing methods physically isolate cells *in vitro* and maintain them in normal cellular physiological conditions. Moreover, many encapsulation methods have been developed in recent years. These techniques are generally classified as either micro-encapsulation or macro-encapsulation [17]. The OEK-induced polymerization principle has potential for applications in cell trapping and encapsulation because of the capsule permeability, mechanical properties, and biocompatibility of hydrogels. Hydrogels have already been used as cell culture scaffolds, and our group reported such work in 2014 [18]. The serial optical images shown in Figure 6a–d show the process of a 5 μm polystyrene bead becoming trapped in a hydrogel tube using the OEK-based polymerization technique. First, a green ring pattern was projected to the OEK chip to trigger the hydrogel polymerization process, which enabled the hydrogel tube to grow and trap the bead. After that, the hydrogel tube continued to elongate and trapped the bead to move away from its original location. The experiment conditions were as follows: voltage was 20 V_pp_ (the equivalent electric field value is 0.4 V_pp_/μm) and the frequency was 10 kHz. The experimental results show that, by using this technique, we can trap and manipulate micro-particles in a liquid solution, potentially paving the way for a technology capable of trapping and manipulating live cells in real time with high spatial resolution. These small hydrogel vesicles could be used to encapsulate particles of different physical sizes, such as proteins, nanoparticles, and cells.

### 3.5. 3D Undercut Features

**Figure 7:**
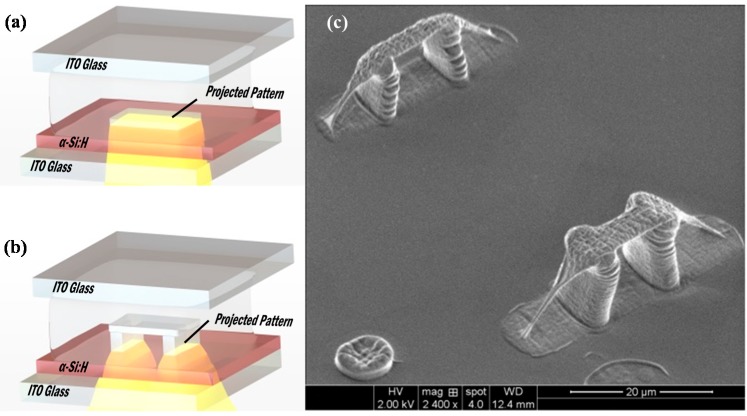
Fabrication process of the 3D suspended micro-scale structure and eSEM image. (**a**,**b**) Schematic of the 3D suspended “microbridge” microstructure fabrication process, the light pattern exposure time in step (**a**) is 10s and in step; (**b**) is 122.5 s; (**c**) eSEM image of PEGDA hydrogel 3D suspended “micro-bridge” structures. The bridge floor thickness is about 2 µm; the height of bridge pillar is about 10 µm.

We demonstrate in this section that the OEK-induced polymerization principle can also be used for 3D micron-scale rapid prototyping, including the fabrication of suspended 3D structures. Suspended, or “undercut” structures can be easily constructed by controlling the image patterns and projection time on the surface of an OEK chip. Figure 7a,b shows the schematic of 3D “micro-bridge” structures fabricated using the OEK-based process. First, a rectangular light pattern of 20 μm × 10 μm in size was projected onto the OEK layer for 10 s to form the “bridge floor.” Then, two small rectangular light patterns of 10 μm × 5 μm in size, with a 10 μm gap between them, were projected onto the OEK chip surface just beneath the bottom of short side of “bridge floor” for 122.5 s to form the “pillars of the bridge”. As the pillars grow upward, the former “bridge floor” could be lifted off by these two pillars. The applied voltage was 20 V_pp_ (the equivalent electric field values was 0.4 V_pp_/μm) with a frequency of 10 kHz for both of these two steps. Figure 7c shows an eSEM image of two hydrogel “bridge” structures fabricated using the OEK-induced polymerization process described above. We note here that the “wave structures” observed in the sidewall of the pillars appeared only after SEM images were taken of the structures, *i.e.* we did not observe these “wave” structures on the pillars under the microscope after the fabrication process. We speculate that these “waves” or “ripples” in the structures are due to the loss of water content inside the hydrogel structures when SEM chamber pumps out air during the vacuuming process. That is, the water content near the surface of the structures evaporate and cause the collapse of the structure, thereby creating “wave-like” feature on the structures.

## 4. Conclusions

We report in this paper a non-UV and non-photoinitiator 3D micron-scale rapid prototyping technique by using an optically induced electrokinetics (OEK) based polymerization process. This new technique allows the fabrication of 3D high-aspect-ratio and suspended hydrogel polymer structures not possible using OEK-based techniques reported in the past. This technique enables the fabrication of micron-scale hydrogel structures of different sizes, shapes, and aspect ratios using a single-step projection process without requiring any microlithographic masks. Furthermore, the rapid OEK-induced polymerization process presented in this paper also enables the “direct writing” of micron-scale hydrogel features within a few seconds to a few minutes, depending on the desired aspect ratio of the microstructures. This OEK-based micro-scale fabrication method achieves a highly parallel fabrication speed by utilizing localized projection light patterns that can be dynamically reconfigured. Using this novel method, hydrogel micropillar arrays rapidly prototyped were used as molds to create micron-scale cavities in PDMS substrates. This method was also used to create hollow, circular tubes with a high-aspect ratio and controllable wall thickness within the time span of a few minutes. Three-dimensional suspended polymer structures were also fabricated, without any sacrificial etching process. In contrast to traditional microlithographic processes, this process does not require relatively expensive and toxic chemicals, such as acetone, methanol or resist-developer; once the microstructures are formed using the OEK-based process, they only need to be rinsed with DI water and dried with nitrogen gas. Hence, in the future, the OEK-based technique could become a very environmentally friendly rapid prototyping technology for the fabrication of polymer microstructures.
